# GSAlign: an efficient sequence alignment tool for intra-species genomes

**DOI:** 10.1186/s12864-020-6569-1

**Published:** 2020-02-24

**Authors:** Hsin-Nan Lin, Wen-Lian Hsu

**Affiliations:** Institute of Information Science, Academia Sinica, Taipei, Taiwan

**Keywords:** Genome comparison, Sequence alignment, Variation detection, Personal genomics, Comparative genomics

## Abstract

**Background:**

Personal genomics and comparative genomics are becoming more important in clinical practice and genome research. Both fields require sequence alignment to discover sequence conservation and variation. Though many methods have been developed, some are designed for small genome comparison while some are not efficient for large genome comparison. Moreover, most existing genome comparison tools have not been evaluated the correctness of sequence alignments systematically. A wrong sequence alignment would produce false sequence variants.

**Results:**

In this study, we present GSAlign that handles large genome sequence alignment efficiently and identifies sequence variants from the alignment result. GSAlign is an efficient sequence alignment tool for intra-species genomes. It identifies sequence variations from the sequence alignments. We estimate performance by measuring the correctness of predicted sequence variations. The experiment results demonstrated that GSAlign is not only faster than most existing state-of-the-art methods, but also identifies sequence variants with high accuracy.

**Conclusions:**

As more genome sequences become available, the demand for genome comparison is increasing. Therefore an efficient and robust algorithm is most desirable. We believe GSAlign can be a useful tool. It exhibits the abilities of ultra-fast alignment as well as high accuracy and sensitivity for detecting sequence variations.

## Background

With the development of sequencing technology, the cost of whole genome sequencing is dropping rapidly. Sequencing the first human genome cost $2.7 billion in 2001; however, several commercial parties have claimed that the $1000 barrier for sequencing an entire human genome is broken [1]. Therefore, it is foreseeable that genome sequencing will become a reality in clinical practices in the near future, which brings the study of personal genomics and comparative genomics. Personal genomics involves the sequencing, analysis and interpretation of the genome of an individual. It can offer many clinical applications, particularly in the diagnosis of genetic deficiencies and human diseases [2]. Comparative genomics is another field to study the genomic features of different organisms. It aims to understand the structure and function of genomes by identifying regions with similar sequences between characterized organisms.

Both personal genomics and comparative genomics require sequence alignment to discover sequence conservation and variation. Sequence conservation patterns can be helpful to predict functional categories, whereas variation can be helpful to infer relationship between organisms or populations in different areas. Studies have shown that variation is important to human health and common genetic disease [3–5]. The alignment speed is an important issue since a genome sequence usually consists of millions of nucleotides or more. Methods based on the traditional alignment algorithms, like AVID [6], BLAST [7] and FASTA [8], are not able to handle large scale sequence alignment. Many genome comparison algorithms have been developed, including ATGC [9, 10], BBBWT [11], BLAT [12], BLASTZ [13], Cgaln [14], chainCleaner [15], Harvest [16], LAGAN [17], LAST [18], MAGIC [19], MUMmer [20–23], and minimap2 [24] .

One of important applications of genome comparison is to identify sequence variations between genomes, which can be found by linearly scanning their alignment result. However, none of the above-mentioned methods have been evaluated the correctness of sequence alignment regarding variation detection. A wrong sequence alignment would produce false sequence variants. In this study, we estimated the performance of each selected genome sequence comparison tool by measuring the correctness of sequence variation. We briefly summarized the algorithm behind each pairwise genome sequence alignment tool in Table S1(Supplementary data). The alignment algorithms can be classified into two groups: seed-and-extend and seed-chain-align, and the seeding schemes can be K-mer, minimizer, suffix tree, suffix array, or BWT.

Recently, many NGS read mapping algorithms use Burrows Wheeler Transformation (BWT) [25] or FM-index [26] to build an index for the reference sequences and identify maximal exact matches by searching against the index array with a query sequence. It has been shown that BWT-based read mappers are more memory efficient than hash table based mappers [27]. In this study, we used BWT to perform seed exploration for genome sequence alignment. We demonstrated that GSAlign is efficient in finding both exact matches and differences between two intra-species genomes. The differences include all single nucleotide polymorphisms (SNPs), insertions, and deletions. Moreover, the alignment is ultra-fast and memory efficient. The source code of GSAlign is available at https://github.com/hsinnan75/GSAlign.

## Implementation

The algorithm of GSAlign is derived from our DNA read mapper, Kart [28]. Kart adopts a divide-and-conquer strategy to separate a read into regions with and without differences. The same strategy is applicable to genome sequence alignment. However, in contrast with NGS short read alignment, genome sequence alignment often consists of multiple sub-alignments that are separated by dissimilar regions or variants. In this study, we present GSAlign for handling genome sequence alignment.

### Algorithm overview

**Fig. 1:**
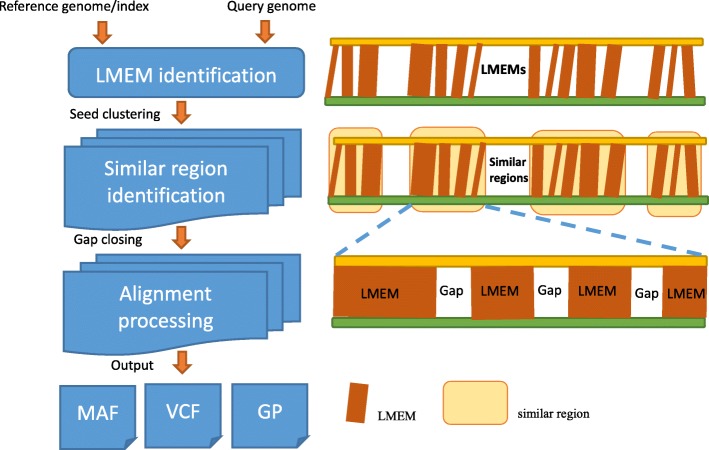
The flowchart of GSAlign. Each rectangle is an LMEM (simple pair) and the width is the size of the LMEM. They are then clustered into similar regions, each of which consists of adjacent LMEMs and gaps in between. We then perform gapped/un-gapped alignment to close those gaps to build the complete alignment for each similar region

Similar to MUMmer4 and Minimap2, GSAlign also follows the “seed-chain-align” procedure to perform genome sequence alignment. However, the details of each step are quite different. Figure 1 illustrates the workflow of GSAlign. It consists of three main steps: *LMEM identification (seed)*, *similar region identification (chain)*, and *alignment processing (align)*. We define a *local maximal exact match* (LMEM) as a common substring between two genomes that begins at a specific position of query sequence. In the LMEM identification step, GSAlign finds LMEMs with variable relengths and then converts those LMEMs into simple pairs. A simple pair represents a pair of identical sequence fragments, one from the reference and one from the query sequence. In the similar region identification, GSAlign clusters those simple pairs into disjoint groups. Each group represents a similar region. GSAlign then finds all local gaps in each similar region. A local gap (defined as a normal pair) is the gap between two adjacent simple pairs. In the alignment-processing step, GSAlign closes gaps to build a complete local alignment for each similar region and identifies all sequence variations during the process. Finally, GSAlign outputs the alignments of all similar regions, a VCF (variant call format) file, and a dot-plot representation (optional). The contribution of this study is that we optimize those steps and integrate them into a very efficient algorithm that saves both time and memory and produces reliable alignments.

### Burrows-Wheeler transform

We give a brief background of BWT algorithm below. Consider a text *T* of length *L* over an alphabet set Σ; *T* is attached with symbol *$* at the end, and *$* is lexicographically smaller than any character in Σ. Let *SA*[0, *L*] be the suffix array of *T*, such that *SA*[*i*] indicates the starting position of the *i*-th lexicographically smallest suffix. The BWT of *T* is a permutation of *T* such that *BWT*[*i*] = *T*[SA[*i*] − 1] (Note that if *SA*[*i*] = 0, *BWT*[*i*] = *$*). Given a pattern *S*, suppose SA[*i*] and SA[*j*] are the smallest and largest suffices of *T* where *P* is their common prefix, the range [*i*, *j*] indicates the occurrences of *S*. Thus, given an SA range [*i*, *j*] of pattern *P*, we can apply the backward search algorithm to find the SA range [*p*, *q*] of *zP* for any character *z*. If we build the BWT with the reverse of *T*, the backward search algorithm can be used to test whether a pattern *P* is an exact substring of *T* in *O*(|*P*|) time by iteratively matching each character in *P*. One of the BWT index algorithms was implemented in BWT-SW [29] and it was then modified to work with BWA [27]. For the details of BWT index algorithm and the search algorithm, please refer to the above-mentioned methods and Kart.

### LMEM identification

Given two genome sequences *P* and *Q*, GSAlign generates the BWT array with *P* and its reverse complementary sequence *P′*. Let *P*[*i*_1_] be the *i*_1_-th nucleobase of *P*, and *P*[*i*_1_, *i*_2_] be the sequence fragment between *P*[*i*_1_] and *P*[*i*_2_]. GSAlign finds LMEMs by searching against the BWT array with *Q*. Since each LMEM is a common substring that begins at a specific position of *Q*, it is represented as a *simple pair* (i.e., identical fragment pair) in this study and denoted by a 4-tuple (*i*_1_, *i*_2_, *j*_1_, *j*_2_), meaning *P*[*i*_1_, *i*_2_] = *Q*[*j*_1_, *j*_2_] and *P*[*i*_2_ + 1] ≠ *Q*[*j*_2_ + 1]. If the common substring appears multiple times (i.e., frequency > 1), it would be transformed into multiple simple pairs. For example, if the substring *Q*[*j*_1_, *j*_2_] is identical to *P*[*i*_1_, *i*_2_] and *P*[*i*_3_, *i*_4_], it would be represented as two simple pairs (*i*_1_, *i*_2_, *j*_1_, *j*_2_) and (*i*_3_, *i*_4_, *j*_1_, *j*_2_). Note that an LMEM is transformed into simple pairs only if its size is not smaller than a user-defined threshold *k* and its occurrences are less than *f*. We investigate the effect of threshold *k* and *f* in the Table S2 (Supplementary data) and we found that GSAlign performs equally well with different thresholds.

The BWT search iteratively matches every nucleotide of the query genome *Q*. It begins with *Q*[*j*_1_] (*j*_1_ = 0 at the first iteration) and stops at *Q*[*j*_2_] if it meets a mismatch at *Q*[*j*_2_ + 1], i.e., the SA range of *Q*[*j*_1_, *j*_2_ + 1] = 0. The next iteration of BWT search will start from *Q*[*j*_2_ + 1] until it meets another mismatch. When GSAlign is running with sensitive mode, the next iteration of BWT search starts from *Q*[*j*_1_ + 5] instead of *Q*[*j*_2_ + 1]. In doing so, GSAlign is less likely to miss true LMEMs due to false overlaps between *P* and *Q*. The search procedure terminates until it reaches the end of genome *Q*.

Please note that the LMEM identification can be processed simultaneously if GSAlign runs with multiple threads. For each query sequence in *Q*, GSAlign divides it into *N* blocks of equal size when it is running with *N* threads and each thread identifies LMEMs for a sequence block independently. The multithreading can be also applied in the following alignment step. We will demonstrate that such parallel processing greatly speedup the alignment process.

### Similar region identification

After collecting all simple pairs, GSAlign sorts all simple pairs according to their position differences between genomes *P* and *Q* and clusters those into disjoint groups. The clustering algorithm is described below.

Suppose *S*_*k*_ is a simple pair (*i*_*k*,1_, *i*_*k*,2_, *j*_*k*,1_, *j*_*k*,2_), we define *PosDiff*_*k*_ = *i*_*k*,1_*− j*_*k*,1_. If two simple pairs have similar *PosDiff*, they are co-linear. We sort all simple pairs according to their *PosDiff* to group all co-linear simple pairs. The clustering starts with the first simple pair *S*_*1*_ and we check if the next simple pair (*S*_*2*_) is within a threshold *MaxDiff* (the default value is 25). The size of *MaxDiff* determines the maximum indel size allowed between two simple pairs. If |*PosDiff*_*1*_ *− PosDiff*_*2*_| ≤ *MaxDiff*, we then check the PosDiff of *S*_*2*_ and *S*_*3*_ until we find two simple pairs *S*_*k*_ and *S*_*k + 1*_ whose |*PosDiff*_*k*_ *− PosDiff*_*k + 1*_| > *MaxDiff*. In such cases, the clustering breaks at *S*_*k + 1*_ and simple pairs *S*_*1*_, *S*_*2*_, …, *S*_*k*_ are clustered in the same group. We investigate the performance of GSAlign with different values of MaxDiff and summarize the analysis in Table S3 (Supplementary data).

We then re-sort *S*_*1*_, *S*_*2*_, …, *S*_*k*_ by their positions at sequence Q (i.e., the third value of 4-tuple). Since simple pairs are re-sorted by their positions at sequence Q, some of them may be not co-linear with their adjacent simple pairs and they are considered as outliers. We remove those outliers from the simple pair group. A simple pair *S*_*m*_ is considered as an outlier if |*PosDiff*_*m*_ *− PosDiff*_*m − 1*_| > 5 and |*PosDiff*_*m*_ *− PosDiff*_*m + 1*_| > 5 where *S*_*m-1*_, *S*_*m*_ and *S*_*m +* 1_ are adjacent. In such cases, we will perform a dynamic programming to handle the gap between *S*_*m-1*_ and *S*_*m + 1*_.

For those simple pairs of same positions at sequence Q (i.e., the fragment of Q has multiple occurrences in P), we keep the one with the minimal difference of *PosDiff* compared to the closest unique simple pair. Then we check every two adjacent simple pairs *s*_*a*_ = (*i*_a,1_, *i*_a,2_, *j*_a,1_, *j*_a,2_) and *s*_*b*_ = (*i*_b,1_, *i*_b,2_, *j*_b,1_, *j*_b,2_), we define gap(*S*_*a*_, *S*_*b*_) = *j*_*b*,1_ *− j*_*a*,2_. If gap(*S*_*a*_, *S*_*b*_) is more than 300 bp and the sequence fragments in the gap are dissimilar, we consider *S*_*b*_ as a break point of a similar region. To determine whether the sequence fragment in a gap are similar, we use k-mers to estimate their similarity. If the number of common k-mers is less than gap(*S*_*a*_, *S*_*b*_) / 3, they are considered dissimilar. In such cases, we consider *S*_*b*_ as a break point of a similar region, and *S*_*b*_ will initiate another similar region. We investigate different gap size thresholds in the Table S4 (Supplementary data) and found that GSAlign was not sensitive to the threshold. The simple pair clustering will be continued with the next un-clustered simple pair until all simple pairs are visited.

**Fig. 2:**
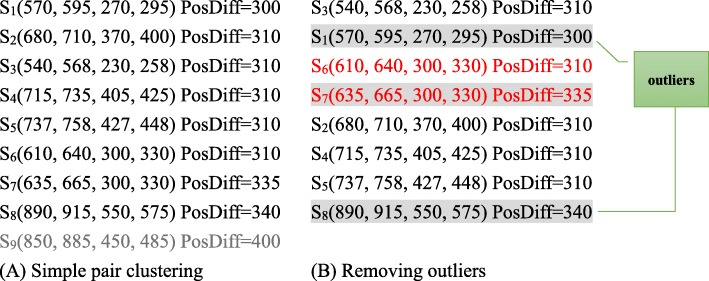
An example illustrating the process of simple clustering and outlier removing. GSAlign clusters simple pairs and remove outliers according to PosDiff. Simple pairs in red are not unique. Simple pairs with gray backgrounds are considered as outliers and they are removed from the cluster

We use an example to illustrate the process of simple pair clustering and outlier removing. Suppose GSAlign identifies nine simple pairs as shown in Fig. 2a. We sort these simple pairs by their PosDiff and start clustering with S_1_. Simple pairs *S*_*1*_, *S*_*2*_, …, *S*_*8*_ are clustered in the same group since any two adjacent simple pairs in the group have similar PosDiff. For example, |*PosDiff*_*1*_ *− PosDiff*_*2*_| = 10, and |*PosDiff*_*2*_ *− PosDiff*_*3*_| = 0. By contrast, |*PosDiff*_*8*_ *− PosDiff*_*9*_| = 60, we break the clustering at *S*_9_. We then re-sort *S*_*1*_, *S*_*2*_, …, *S*_*8*_ by their positions at sequence Q as shown in Fig. 2b, and mark S_6_ and S_7_ are not unique since the two simple pairs have the same position at Q. We compare S_6_ and S_7_ and keep S_6_ because it has the minimal difference of *PosDiff* with its neighboring unique simple pairs.

We remove S_1_ and S_8_ since they are not co-linear with their adjacent simple pairs. S_1_ is considered an outlier because |*PosDiff*_*1*_ *− PosDiff*_*3*_| > 5 and |*PosDiff*_*1*_ *− PosDiff*_*6*_| > 5. After S_1_ is removed, the gap between S_3_ and S_6_ would probably form an un-gapped alignment since they have the same PosDiff. S_8_ is also an outlier because |*PosDiff*_*8*_ *− PosDiff*_*5*_| > *MaxDiff*. Finally, we confirm there is no any large gap between any two adjacent simple pairs in the group. Thus, the group of S_3_, S_6_, S_2_, S_4_, and S_5_ forms a similar region, and upon which we can generate a local alignment.

**Fig. 3:**
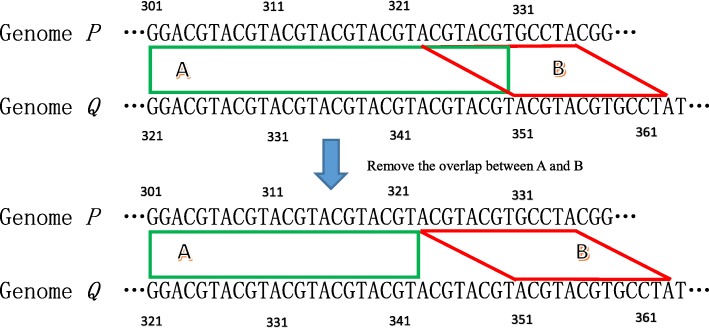
Simple pairs **a** and **b** overlaps due to tandem repeats of “ACGT”. We remove the overlapped fragment from simple pair A (the preceding one)

Given two adjacent simple pairs in the same cluster, *s*_*a*_ = (*i*_a,1_, *i*_a,2_, *j*_a,1_, *j*_a,2_) and *s*_*b*_ = (*i*_b,1_, *i*_b,2_, *j*_b,1_, *j*_b,2_), we say *s*_*a*_ and *s*_*b*_ overlap if *i*_a,1_ ≤ *i*_b,1_ ≤ *i*_a,2_ or *j*_a,1_ ≤ *j*_b,1_ ≤ *j*_a,2_. In such cases, the overlapping fragment is chopped off from the smaller simple pair. For example, BWT index. Figure 3. shows a tandem repeat with different copies in genome *P* and *Q*. In this example, “ACGT” is a tandem repeat where *P* has seven copies and *Q* has nine copies. GSAlign identifies two simple pairs in this region: *A* (301, 330, 321, 350) and *B* (323, 335, 351, 363). *A* and *B* overlap between *P*[323, 330]. In such cases, we remove the overlap from the preceding simple pair (i.e., *A*). After removing the overlap, *A* becomes (301, 322, 321, 342) and we create a gap of *Q*[343, 350]. After removing overlaps, we check if there is a gap between any two adjacent simple pairs in each similar region. We fill gaps by inserting normal pairs. A normal pair is also denoted as a 4-tuple (*i*_1_, *i*_2_, *j*_1_, *j*_2_) in which *P*[*i*_1_, *i*_2_] ≠ *Q*[*j*_1_, *j*_2_] and the size of *P*[*i*_1_, *i*_2_] or *Q*[*j*_1_, *j*_2_] can be 0 if one of them is an deletion. Suppose we are given two adjacent simple pairs (*i*_2*q*-1_, *i*_2*q*_, *j*_2*q*-1_, *j*_2*q*_) and (*i*_2*q* + 1_, *i*_2*q +* 2_, *j*_2*q* + 1_, *j*_2*q* + 2_). If *i*_2*q* + 1_ − *i*_2*q*_ > 1 or *j*_2*q* + 1_ − *j*_2*q*_ > 1, then we insert a normal pair (*i*_*r*_, *i*_*r* + 1_, *j*_*r*_, *j*_*r* + 1_) to fill the gap, where *i*_*r*_ – *i*_2*q*_ = *i*_2*q* + 1_ – *i*_*r* + 1_ = 1 if *i*_2*q* + 1_ − *i*_2*q*_ > 1; otherwise *i*_*r*_ = *i*_*r* + 1_ = − 1 meaning the corresponding fragment size is 0. Likewise, *j*_*r*_ – *j*_2*q*_ = *j*_2*q* + 1_ – *j*_*r* + 1_ = 1 if *j*_2*q* + 1_ − *j*_2*q*_ > 1, otherwise let *j*_*r*_ = *j*_*r* + 1_ = − 1.

### Alignment processing

At this point, GSAlign has identified similar regions that consist of simple pairs and normal pairs. In this step, GSAlign only focuses on normal pairs. If the sequence fragments in a normal pair have equal size, it is very likely the sequence fragments only contain substitutions and the un-gapped alignment is already the best alignment; if the sequence fragments contain indels, gapped alignment is required. Therefore, we classify normal pairs into the following types:

1) A normal pair is *Type I* if the fragment pair has equal size and the number of mismatches in a linear scan is less than a threshold;

2) A normal pair is *Type II* if one of the fragment is a null string and the other contains at least one nucleobase;

3) The remaining normal pairs are *Type III;*

Thus, only *Type III* require gapped alignment. GSAlign applies the KSW2 algorithm [30] to perform gapped alignment. The alignment of each normal pair is constrained by the sequence fragment pair. This allows GSAlign to generate their alignments simultaneously with multiple threads. At the end, the complete alignment of the genome sequences is the concatenation of the alignment of each simple and normal pairs.

### Differences among GSAlign, MUMmer4, and Minimap2

In general, GSAlign, MUMmer4, and Minimap2 follow the conventional seed-chain-align procedure to align genome sequences. However, the implementation details are very different from each other. MUMmer4 combines the ideas of suffix arrays, the longest increasing subsequence (LIS) and Smith-Waterman alignment. Minimap2 uses minimizers (k-mers) as seeds and identifies co-linear seeds as chains. It applies a heuristic algorithm to cluster seeds into chains and it uses dynamic programming to closes between adjacent seeds. GSAlign integrates the ideas of BWT arrays, PosDiff-based clustering and dynamic programming algorithm. GSAlign divides the query sequence into multiple blocks and identifies LMEMs on each block simultaneously using multiple threads. More importantly, GSAlign classifies normal pairs into three types and only *Type III* normal pairs require gapped alignment. This divide-and-conquer strategy not only reduces the number of fragment pairs requiring gapped alignment, but also shortens gap alignment sizes. Furthermore, GSAlign can produce the alignments of normal pairs simultaneously with multi-threads. Though MUMmer4 supports multi-threads to align query sequences in parallel, the concurrency is restricted to the number of sequences in the query.

## Results

### Experiment design

GSAlign takes two genome sequences: one is the reference genome for creating the BWT index, and the other is the query genome for searching against the BWT array. If the reference genome has been indexed beforehand, GSAlign can read the index directly. After comparing the genome sequences, GSAlign outputs all local alignments in MAF format or BLAST-like format, a VCF file, and a dot-plot representation (optional) for each query sequence.

The correctness of sequence alignment is an important issue and variant detection is one of the major applications for genome sequence alignment. Therefore, we estimate the correctness of sequence alignments by measuring the variant detection accuracy. Though most of genome alignment tools do not output variants, we can identify variants by linearly scanning the sequence alignments. This measurement is sensitive to misalignments; thus we consider it is a fair measurement to estimate the performance of sequence alignment.

We randomly generate sequence variations with the frequency of 20,000 substitutions (SNVs), 350 small indels (1~10 bp), 100 large indels (11~20 bp) for every 1 M base pairs. To increase the genetic distance, we generate different frequencies of SNVs. Benchmark datasets labelled with 1X contain around 20,000 SNVs for every 1 M base pairs, whereas datasets labelled with 3X (or 5X) contain 60,000 (or 100,000) SNVs per million bases. We generate three synthetic datasets with different SNV frequencies using the human genome (GRCh38). The synthetic datasets are referred to as simHG-1X, simHG-3X, and simHG-5X, respectively. To evaluate the performance of genome sequence alignment on real genomes, we download the diploid sequence of NA12878 genome and its reference variants (the sources are shown in Supplementary data). We also estimate the Average Sequence Identity (ASI) based on the total number of mismatches due to the sequence variants over the genome size. For example, an SNV event produce one mismatch and an indel event of size *n* produces *n* mismatches. Thus, the ASI of the four datasets are 97.93, 93.86, 89.90, and 99.84%, respectively.

The diploid sequence of NA12878 consists of 3,088,156 single nucleotide variants (SNVs) and 531,315 indels. The reference variants are generated from NGS data analysis. Please note that GSAlign is a genome alignment tool, rather than a variant caller such as Freebayes or GATK HaplotypeCaller (GATK-HC). GSAlign identifies variants from genome sequence alignment, while Freebayes and GATK-HC identify variants from NGS short read alignments. We use sequence variants to estimate the correctness of sequence alignment in this study. Table 1 shows the genome size, the variant numbers of SNV, small and large indels as well as the ASI of each benchmark dataset.

**Table 1:** The synthetic datasets and the number of simulated sequence variations. The Average Sequence Identity (ASI) is estimated by the total mismatches divided by the number of nucleobases

Dataset	Genome size	SNV	Small indel	large indel	ASI
simHG-1X	3,088,279,342	58,421,383	1,001,626	285,757	97.93%
simHG-3X	3,088,292,247	175,100,939	962,721	275,584	93.86%
simHG-5X	3,088,289,999	291,714,646	919,762	263,271	89.90%
NA12878	6,070,700,436	3,088,156	531,315	NA	99.84%

In this study, we compare the performance of GSAlign with several existing genome sequence aligners, including LAST (version 828), Minimap2 (2.17-r943-dirty), and MUMmer4 (version 4.0.0beta2). We exclude the others because they are either unavailable or developed for multiple sequence alignments, like Cactus [31], Mugsy [32], or MULTIZ [33]. We exclude BLAT because it fails to produce alignments for larger sequence comparison; we exclude LASTZ because it does not support multi-thread. Moreover, LASTZ fails to handle human genome alignment.

### Measurement

We define true positives (TP) as those variants which are correctly identified from the sequence alignment; false positives (FP) as those variants which are incorrectly identified; and false negatives as those true variants which are not identified. A predicted SNV event is considered true if the genomic coordinate is exactly identical to the true event; a predicted indel event is considered true if the predicted coordinate is within 10 nucleobases of the corresponding true event. The precision and recall are defined as follows: precision = TP / (TP + FP) and recall = TP / (TP + FN).

To estimate the performance for existing methods, we filter out sequence alignments whose sequence identity are lower than a threshold (for Mummer4 and LAST) or those whose quality score are 0 (for Minimap2). The argument setting used for each method is shown in the Table S5 (Supplementary data). We estimate the precision and recall on the identification of sequence variations for each dataset. GSAlign, Minimap2, MUMmer4, and LAST can load premade reference indexes; therefore, we run these methods by feeding the premade reference indexes and they are running with 8 threads.

### Performance evaluation on synthetic datasets

Table 2 summarizes the performance result on the three synthetic datasets. It is observed that GSAlign and Minimap2 have comparable performance on the benchmark dataset. Both produce alignments that indicate sequence variations correctly. MUMmer4 and LAST produce less reliable alignments than GSAlign and Minimap2. Though we have filtered out some of alignments based on sequence identity, their precisions and recalls are not as good as those of GSAlign and Minimap2. In particular, the precision of indel events of MUMmer4 and LAST are much lower on the dataset of simHG-5X. It implies that the two methods are not designed for genome sequence alignments with less sequence similarity. We also compare the total number of local alignments each method produces for the benchmark datasets. It is observed that GSAlign produces the least number of local alignments, though it still covers most of the sequence variants. For example, GSAlign produces 250 local alignments for simHG-1X, whereas the other three methods produce 417, 3111 and 1168 local alignments, respectively.

**Table 2:** The performance evaluation on the three GRCh38 synthetic data sets. The indexing time of each method is not included in the run time. They are 110 (BWT-GSAlign), 129 (Suffix array-MUMmer4), and 2.6 min (Minimizer-Minimap2), respectively

Dataset	Method	SNV		Indel		Local align#	Run time (min)
		precision	recall	precision	recall		
SimHG-1X	GSAlign	1.000	1.000	0.999	0.999	250	11
	Minimap2	1.000	0.996	0.999	0.995	417	39
	MUMmer4	0.998	0.932	0.985	0.932	3111	869
	LAST	1.000	0.992	0.992	0.947	1168	2524
SimHG-3X	GSAlign	1.000	0.998	0.994	0.997	366	18
	Minimap2	1.000	0.996	0.991	0.995	561	37
	MUMmer4	0.989	0.923	0.796	0.925	4925	289
	LAST	1.000	0.990	0.809	0.950	1234	1185
SimHG-5X	GSAlign	1.000	0.993	0.958	0.992	587	24
	Minimap2	1.000	0.995	0.952	0.994	1058	40
	MUMmer4	0.986	0.907	0.486	0.912	5513	157
	LAST	1.000	0.981	0.461	0.947	1636	458

In terms of runtime, it can be observed that GSAlign spends the least amount of runtime on the three datasets. Minimap2 is the second fastest method. Though MUMmer4 is faster than LAST, it produces worse performance than LAST. We observe that LAST is not very efficient with multi-threading. Though it runs with eight threads, it only uses single thread most of time during the sequence comparison. Interestingly, GSAlign spends more time on less similar genome sequences (ex. simHG-5X) because there are more gapped alignments, whereas MUMmer4 and LAST spends more time on more similar genome sequences (ex. simHG-1X) because they handle more number of seeds. Minimap2 spends similar amount of time on the three synthetic datasets because Minimap2 produces similar number of seeds for those datasets. Note that it is possible to speed up the alignment procedure by optimizing the parameter settings for each method; however, it may complicate the comparison.

### Performance evaluation on NA12878

The two sets of diploid sequence of NA12878 are aligned separately and the resulting VCF files are merged together for performance evaluation. Because many indel events of NA12878 locate in tandem repeat regions, we consider a predicted indel is a true positive case if it locates at either end of the repeat region. For example, the two following alignments produce identical alignment scores:

AGCATGCATTG AGCATGCATTG.

AGCAT----TG, and AG----CATTG.

It can be observed that the two alignments produce different indel events.

In such case, both indel events are considered true positives if one of them is a true indel.

Table 3 summaries the performance evaluation on the real dataset. It is observed that GSAlign, Minimap2 and LAST produce comparable results on SNV and indel detection. They have similar precisions and recalls. However, their precisions and recalls are much worse than those on synthetic datasets. It seems counter-intuitive since the synthetic datasets contain much more variants than NA12878 genomes. Thus, we reconstruct the NA12878 genome sequence directly from the reference variants and call variants using GSAlign. The precision and recall on SNV detection become 0.996 and 0.998 and those on indel detection become 0.994 and 0.983. It implies that the diploid genome sequence and the reference variants are not fully compatible.

**Table 3:** The performance evaluation on HG38 and the diploid sequence of NA12878. The performance on SNV and Indel detection implies that the diploid genome sequence and the reference variants are not fully compatible

Dataset	Method	SNV		Indel		Run time (min)	Memory usage (GB)
		Precision	Recall	Precision	Recall		
NA12878 (Diploid)	GSAlign	0.832	0.969	0.759	0.767	5	14
	Minimap2	0.830	0.970	0.754	0.768	65	23
	MUMmer4	0.752	0.946	0.711	0.749	3898	57
	LAST	0.832	0.969	0.760	0.764	1305	28

In terms of runtime, it is observed that GSAlign only spends 5 min to align the diploid sequences of NA12878 with HG38. Minimap2 is the second fastest method. It spends 65 min. LAST and MUMmer4 spend 1305 and 3898 min, respectively. In terms of memory consumption, it is observed that GSAlign consumes the least amount of memory among the selected methods. It requires 14 GB to perform the genome comparison, while MUMmer4 requires 57 GB.

## Discussion

### Sequence comparison between difference species

Though GSAlign is designed for comparing intra-species genomes, it can be used to identify conserved syntenic regions for inter-species genomes. Here we compare human genomes with whole chimpanzee genome and mouse chromosome 12. We compare human (GRCh38) and chimpanzee (PanTro4) genomes using the four selected tools with 8 threads. Since the ground-truth alignment between GRCh38 and chimpanzee (PanTro4) genomes is unknown, we only show the total alignment length, the predicted SNV and Indel numbers of each alignment tool as well as their run time. We summarize their result in Table 4. Though the genome size PanTro4 is around 3146.6 Mbp, it contains around 2757.6 M known nucleotides. GSAlign spends eight minutes on the genome comparison and generates alignments of total length 2412 Mbp. Minimap2 is the second fastest method and it generates alignments of total length 2791 Mbp. LAST and MUMmer4 are much slower. They generate alignments of total length 2717 and 2661 Mbps, respectively.

**Table 4:** The performance comparison on HG38 and the chimpanzee (PanTro4) genome

Dataset	Method	Alignment length (Mbp)	SNV#	Indel#	Run time (min)
GRCh38 Vs. PanTro4	GSAlign	2412	31,710,527	3,650,337	8
	Minimap2	2791	39,242,895	4,375,360	18
	MUMmer4	2661	41,545,986	5,450,956	1368
	LAST	2717	35,815,610	4,483,929	884

**Fig. 4:**
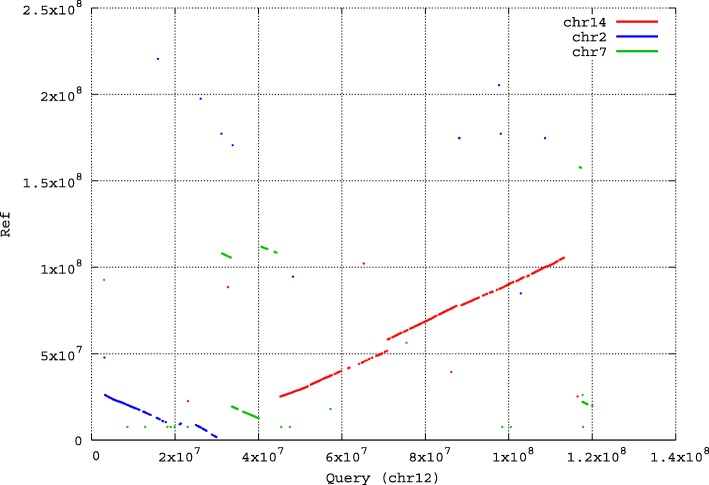
The dot-plot of the alignment for human chromosomes 2, 7, and 14 and mouse chromosome 12. The x-axis indicates the positions of mouse chromosome 12, and y-axis indicates the positions of human chromosomes 2, 7 and 14. The orthologous landmarks are plotted based on the pairwise alignments between the three human chromosomes and mouse chromosome 12

Mouse chromosomes share common ancestry with human chromosomes [34]. Here we demonstrate the sequence comparison between human genome and mouse chromosome 12 by showing the dot-plot matrix generated by GSAlign. Though the genome sequences of the two species are very dissimilar, they still share conservation of genetic linkage groups. In this analysis, GSAlign spends three minutes to compare HG38 and mouse chromosome 12 and it generates 2713 local alignments with a total length of 1738 K bases. Among the 22 body human chromosomes, GSAlign discovers that human chromosomes 2, 7 and 14 share the largest number of conserved syntenic segments with mouse chromosome 12. GSAlign visualizes the conserved syntenic segments with a dot-plot presentation in Fig. 4. The x-axis indicates the positions of mouse chromosome 12, and y-axis indicates the positions of human chromosomes 2, 7 and 14. The orthologous landmarks are plotted based on the pairwise alignments between the three human chromosomes and mouse chromosome 12. Comparing the result with existing studies, we find that the dot-plot is consistent with Fig. 4f in the study of Mouse Genome Sequencing Consortium [34].

## Conclusions

In this study, we present GSAlign, a new alignment tool that handles genome sequence comparison. We evaluate the correctness of sequence alignment by measuring the accuracy of variant detection. GSAlign adopts the divide-and-conquer strategy to divide genome sequences into gap-free fragment pairs and gapped fragment pairs. GSAlign is a BWT-based genome sequence aligner. Therefore, it requires less amount of memory than hash table-based or tree-based aligners do. GSAlign also supports multi-thread computation, thus it is more efficient when comparing large genomes. We evaluate the performances of GSAlign with synthetic and real datasets. The experiment result shows that GSAlign is the fastest among the selected methods and it produces perfect or nearly perfect precisions and recalls on the identification of sequence variations for most of the datasets. We also found that the diploid genome sequence of NA12878 is not fully compatible with the reference variants derived from NGS data.

As more genome sequences become available, the demand for genome comparison is increasing. Therefore, an efficient and robust algorithm is most desirable. We believe GSAlign can be a useful tool. It shows the abilities of ultra-fast alignment as well as high accuracy and sensitivity for detecting sequence variations.

## Availability and requirements

Project name: GSAlign.

Project home page: https://github.com/hsinnan75/GSAlign

Operating system: Linux.

Programming language: C/C++.

Other requirements: N/A.

License: MIT License.

## Supplementary information

**Additional file 1 **: **Table S1**. A summary of several existing genome sequence alignment tools. **Table S2**. The effect of minimal LMEM size k and their maximal frequency f for GSAlign on the Sim_Chr1 dataset. **Table S3**. The effect of MaxPosDiff for GSAlign on the Sim_Chr1 dataset. **Table S4**. The effect of gap size threshold on the Sim_Chr1 dataset. **Table** ***S5*** lists the argument setting for each method tested in this study. Aligner and their arguments used on the benchmark datasets, where fa1 and fa2 are input genomes with FASTA format.

## Data Availability

The datasets supporting the conclusions of this article are available at http://bioapp.iis.sinica.edu.tw/~arith/GSAlign/.

## Abbreviations

ASI: Average Sequence Identity
BWT: Burrows Wheeler Transformation
FN: False negative
FP: False positive
LMEM: Local maximal exact match
SNV: Single Nucleotide Variation
TP: True positive
VCF: Variant call format

## References

[1] van Ninnwegen KJM, van Soest RA, Veltman JA, Nelen MR, van der Wilt GJ, Vissers LELM, Grutters JPC (2016). Is the $1000 Genome as near as we think? A cost analysis of next-generation Sequencing. Clin Chem.

[2] Roberts NJ, Vogelstein JT, Parmigiani G, Kinzler KW, Vogelstein B, Velculescu VE (2012). The predictive capacity of personal genome sequencing. Sci Transl Med.

[3] Sudmant PH, Rausch T, Gardner EJ, Handsaker RE, Abyzov A, Huddleston J, Zhang Y, Ye K, Jun G, Hsi-Yang Fritz M (2015). An integrated map of structural variation in 2,504 human genomes. Nature.

[4] Feuk L, Carson AR, Scherer SW (2006). Structural variation in the human genome. Nat Rev Genet.

[5] Pang AW, MacDonald JR, Pinto D, Wei J, Rafiq MA, Conrad DF, Park H, Hurles ME, Lee C, Venter JC (2010). Towards a comprehensive structural variation map of an individual human genome. Genome Biol.

[6] Bray N, Dubchak I, Pachter L (2003). AVID: a global alignment program. Genome Res.

[7] Altschul SF, Gish W, Miller W, Myers EW, Lipman DJ (1990). Basic local alignment search tool. J Mol Biol.

[8] Pearson WR, Lipman DJ (1988). Improved tools for biological sequence comparison. Proc Natl Acad Sci U S A.

[9] del Cuvillo J, Tian XM, Gao GR, Girkar M (2003). Performance study of a whole genome comparison tool on a hyper-threading multiprocessor. High Perform Comput.

[10] Martins WS, Cuvillo J, Cui W, Gao GR. Whole genome alignment using a multithreaded parallel implementation. Pirenopolis: Symposium on Computer Architecture and High Performance Computing; 2001. p. 1–8.

[11] Lippert RA (2005). Space-efficient whole genome comparisons with Burrows-Wheeler transforms. J Comput Biol.

[12] Kent WJ (2002). BLAT--the BLAST-like alignment tool. Genome Res.

[13] Schwartz S, Kent WJ, Smit A, Zhang Z, Baertsch R, Hardison RC, Haussler D, Miller W (2003). Human-mouse alignments with BLASTZ. Genome Res.

[14] Nakato R, Gotoh O (2010). Cgaln: fast and space-efficient whole-genome alignment. BMC Bioinform.

[15] Suarez HG, Langer BE, Ladde P, Hiller M (2017). ChainCleaner improves genome alignment specificity and sensitivity. Bioinformatics.

[16] Treangen TJ, Ondov BD, Koren S, Phillippy AM (2014). The harvest suite for rapid core-genome alignment and visualization of thousands of intraspecific microbial genomes. Genome Biol.

[17] Brudno M, Do CB, Cooper GM, Kim MF, Davydov E, Program NCS, Green ED, Sidow A, Batzoglou S (2003). LAGAN and multi-LAGAN: efficient tools for large-scale multiple alignment of genomic DNA. Genome Res.

[18] Kielbasa SM, Wan R, Sato K, Horton P, Frith MC (2011). Adaptive seeds tame genomic sequence comparison. Genome Res.

[19] Swidan F, Rocha EP, Shmoish M, Pinter RY (2006). An integrative method for accurate comparative genome mapping. PLoS Comput Biol.

[20] Delcher AL, Kasif S, Fleischmann RD, Peterson J, White O, Salzberg SL (1999). Alignment of whole genomes. Nucleic Acids Res.

[21] Delcher AL, Phillippy A, Carlton J, Salzberg SL (2002). Fast algorithms for large-scale genome alignment and comparison. Nucleic Acids Res.

[22] Kurtz S, Phillippy A, Delcher AL, Smoot M, Shumway M, Antonescu C, Salzberg SL (2004). Versatile and open software for comparing large genomes. Genome Biol.

[23] Marcais G, Delcher AL, Phillippy AM, Coston R, Salzberg SL, Zimin A (2018). MUMmer4: A fast and versatile genome alignment system. PLoS Comput Biol.

[24] Li H (2018). Minimap2: pairwise alignment for nucleotide sequences. Bioinformatics.

[25] Burrows M, Wheeler DJ: A block-sorting lossless data compression algorithm**.** 1994.

[26] Ferragina P, Manzini G: Opportunistic data structures with applications**.** University of Pisa; 2000.

[27] Li H, Durbin R (2009). Fast and accurate short read alignment with Burrows-Wheeler transform. Bioinformatics.

[28] Lin HN, Hsu WL (2017). Kart: a divide-and-conquer algorithm for NGS read alignment. Bioinformatics.

[29] Lam TW, Sung WK, Tam SL, Wong CK, Yiu SM (2008). Compressed indexing and local alignment of DNA. Bioinformatics.

[30] Suzuki H, Kasahara M. Introducing difference recurrence relations for faster semi-global alignment of long sequences. BMC Bioinformatics. 2018;19;19(Suppl 1):45. 10.1186/s12859-018-2014-8 PMC5836832 29504909

[31] Paten B, Earl D, Nguyen N, Diekhans M, Zerbino D, Haussler D (2011). Cactus: algorithms for genome multiple sequence alignment. Genome Res.

[32] Angiuoli SV, Salzberg SL (2011). Mugsy: fast multiple alignment of closely related whole genomes. Bioinformatics.

[33] Blanchette M, Kent WJ, Riemer C, Elnitski L, Smit AFA, Roskin KM, Baertsch R, Rosenbloom K, Clawson H, Green ED (2004). Aligning multiple genomic sequences with the threaded blockset aligner. Genome Res.

[34] Mouse Genome Sequencing C, Waterston RH, Lindblad-Toh K, Birney E, Rogers J, Abril JF, Agarwal P, Agarwala R, Ainscough R, Alexandersson M (2002). Initial sequencing and comparative analysis of the mouse genome. Nature.

