# Supplementary material for: GSAlign: an efficient sequence alignment tool for intra-species genomes
Source: BMC Genomics. 2020 Feb 24;21:182. doi: 10.1186/s12864-020-6569-1 (PMC7041101; doi:10.1186/s12864-020-6569-1)
Supplement: Supplementary file 1 — Additional file 1 : Table S1. A summary of several existing genome sequence alignment tools. Table S2. The effect of minimal LMEM size k and their maximal frequency f for GSAlign on the Sim_Chr1 dataset. Table S3. The effect of MaxPosDiff for GSAlign on the Sim_Chr1 dataset. Table S4. The effect of gap size threshold on the Sim_Chr1 dataset. Table S5 lists the argument setting for each method tested in this study. Aligner and their arguments used on the benchmark datasets, where fa1 and fa2 are input genomes with FASTA format. [file 12864_2020_6569_MOESM1_ESM.docx]

GSAlign – an efficient sequence alignment tool for intra-species genomes

Hsin-Nan Lin and Wen-Lian Hsu

Institute of Information Science, Academia Sinica, Taipei, Taiwan

Supplementary Data

1. A summary of existing genome sequence alignment tools

Table S1. A summary of several existing genome sequence alignment tools

| Method | Algorithm | Seeding | Description |
| --- | --- | --- | --- |
| AVID | seed-chain-align | suffix tree | AVID finds maximal matches between two sequences using a suffix tree structure. It then clusters all the matches and split the sequences accordingly. Each cluster forms a local alignment of the two input sequences. |
| BBBWT | seed-match | BWT | BBBWT only finds all bi-unique *k*-mers in common between two genomes. |
| BLAT | seed-and-extend | K-mer | BLAT rapidly scans for relatively short matches and extends those into high-scoring pairs. |
| BLASTZ | seed-and-extend | K-mer | BLASTZ first finds short near-exact matches, extends each short match without allowing gaps, and then extends these again by a dynamic programming procedure that permits gaps. |
| Cgaln | seed-and-extend | K-mer | Cgaln divides sequences into blocks with a fixed length and performs block-to-block similarity evaluation that is based on the frequency of common seeds in the blocks. The nucleotide-level alignment is derived from the block-level alignments. |
| Harvest | seed-chain-align | suffix tree | Harvest identifies maximal unique matches (MUMs) and uses MUMs to recruit similar genomes and anchor the multiple alignment. |
| LAST | seed-and-extend | K-mer | LAST uses adaptive seeds to increase both alignment sensitivity and speed for large sequence comparison. Adaptive seeds are matches that are chosen based on their rareness. |
| LASTZ | seed-and-extend | K-mer | LASTZ is a drop-in replacement for BLASTZ. It adopts similar alignment procedure, though it includes more seeding strategies and reduces memory requirements. |
| MUMmer | seed-chain-align | suffix array | MUMmer identifies all maximal matches between the two se-quences and then finds the longest increasing subsequence (LIS) from the sorted matches. The pairwise sequence alignment is built on the LIS by performing the Smith-Waterman alignment to close gaps between the ordered matches. |
| Minimap2 | seed-chain-align | minimizer | Minimap2 collects minimizers of the reference sequences and creates a hash table to index those minimizers. Then it finds all query minimizers to find exact matches to the reference, and identifies co-linear anchors as chain. Mimimap2 applies dynamic programming to extend from the ends of chains and to close gaps between adjacent anchors in chains. |

2. The effect of minimal LMEM size *k* and their maximal frequency *f*

The minimal LMEM size *k* is a threshold to determine whether an LMEM is considered a qualified seed and the maximal frequency *f* is another threshold to filter out LMEMs that appear too frequently. A small *k* will introduce many false seeds, whereas a large *k* will lose true seeds when they include sequence variations. Likewise, a small *f* will filter out some seeds with higher frequency and increase the genome comparison speed; however, it may lose the alignments at the repetitive regions. The default values of *k* and *f* in GSAlign are 20 and 100. We used default values to estimate the performance of GSAlign on all benchmark datasets. Here, we change the values of *k* and *f* to investigate the effect of the two parameters in terms of run time and variant detection performance on the dataset of Sim_Chr1. Table *S1* summaries the analysis result. We tested *k* with 10, 15, and 20, and we also tested *f* with 100, 300, and 500. In this analysis, GSAlign used a pre-built index to speed up the process. It can be observed that GSAlign performs similarly with different values of *k* and different values of *f* did not affect the performance on variation detection, either.

Table S2. The effect of minimal LMEM size k and their maximal frequency f for GSAlign on the Sim_Chr1 dataset.

| Sim_Chr1 | k / f values | Precision | | Recall | | | | Run Time  (seconds) |
| --- | --- | --- | --- | --- | --- | --- | --- | --- |
|  |  | Sub | Indel | Sub | Indel | SV | CNV |  |
|  | 10 / 100 | 1.000 | 0.990 | 1.000 | 0.991 | 0.989 | 1.000 | 125 |
|  | 15 / 100 | 1.000 | 0.990 | 1.000 | 0.991 | 0.989 | 1.000 | 148 |
|  | 20 / 100 | 1.000 | 0.990 | 1.000 | 0.991 | 0.987 | 1.000 | 134 |
|  | 20 / 300 | 1.000 | 0.990 | 1.000 | 0.991 | 0.987 | 1.000 | 167 |
|  | 20 / 500 | 1.000 | 0.990 | 1.000 | 0.991 | 0.987 | 1.000 | 165 |

3. The effect of MaxPosDiff threshold

The size of MaxPosDiff determines the maximum indel size is allowed between adjacent seeds (simple pairs). We separate two adjacent simple pairs *S_a_* and *S_b_* into separate clusters if |*PosDiff_a_* *− PosDiff_b_*| ≥ *MaxPosDiff*. The default value of MaxPosDiff is 25. Here, we investigate the effect of MaxPosDiff. Table *S3* summaries the analysis result. We tested MaxPosDiff between 25 and 50 on Sim_Chr1. It can be observed that GSAlign performs equally well with different thresholds.

Table S3. The effect of MaxPosDiff for GSAlign on the Sim_Chr1 dataset.

| Sim_Chr1 | MaxPosDiff | SNV | | Indel | | Run time  (second) |
| --- | --- | --- | --- | --- | --- | --- |
|  |  | Precision | Recall | Precision | Recall |  |
|  | 25 | 1.000 | 1.000 | 0.998 | 0.997 | 39 |
|  | 35 | 1.000 | 1.000 | 0.998 | 0.997 | 43 |
|  | 50 | 1.000 | 1.000 | 0.998 | 0.997 | 45 |

4. The effect of gap size threshold between simple pairs

Suppose two adjacent simple pairs *s_a_* = (*i*_a,1_, *i*_a,2_, *j*_a,1_, *j*_a,2_) and *s_b_* = (*i*_b,1_, *i*_b,2_, *j*_b,1_, *j*_b,2_), we define gaps(*S_a_*, *S_b_*) = *j_b_*_,1_ *−* *j_a_*_,2_. If gaps(*S_a_*, *S_b_*) is more than 300bp and the sequences in the gaps are dissimilar, the two simple pairs will be separated into different groups. Here, we investigate the effect of different gap sizes on Sim_Chr1. Table S4 summaries the result. It can be observed that the three different gap sizes performed equally well in terms of variant detection.

Table S4. The effect of gap size threshold on the Sim_Chr1 dataset.

| Sim_Chr1 | Gap size | SNV | | Indel | | Run time  (second) |
| --- | --- | --- | --- | --- | --- | --- |
|  |  | Precision | Recall | Precision | Recall |  |
|  | 100 | 1.000 | 1.000 | 0.998 | 0.997 | 41 |
|  | 300 | 1.000 | 1.000 | 0.998 | 0.997 | 39 |
|  | 500 | 1.000 | 1.000 | 0.998 | 0.997 | 41 |

5. The selected genome comparison tools and their argument setting

Table *S5* lists the argument setting for each method tested in this study.

| Genome comparison tool | Arguments |
| --- | --- |
| GSAlign | GSAlign -i *idx* -q query.fa -t 8 (for benchmark datasets and PanTro4)  GSAlign -i *idx* -q query.fa -sen -t 8 (for mouse chromosome 12) |
| Minimap2 | minimap2 -d *idx* ref.fa  minimap2 -t 8 -ax asm10 *idx* fa2 > out.sam (for simHG-1x and simHG-3x)  minimap2 -t 8 -ax asm20 *idx* fa2 > out.sam (for simHG-5x) |
| MUMmer4 | nucmer --mum --threads=8 --load=*idx* --prefix=out fa1 fa2 (for benchmark datasets)  delta-filter -q out.delta > out.filter.delta  delta2maf out.filter.delta > out.maf |
| LAST | lastdb -uNEAR -R01 *idx* fa1  lastal -P8 -l30 -k20 -E0.01 *idx* fa2 \| last-split –m1 > out.maf |

Table S5. Aligner and their arguments used on the benchmark datasets, where fa1 and fa2 are input genomes with FASTA format.

6. Benchmark datasets and sequence mutation simulator

The sequence mutation simulation (SVsim.cpp), benchmark datasets, and evaluation program (Evaluation.cpp) in this study are available at <http://bioapp.iis.sinica.edu.tw/~arith/GSAlign/>.

The diploid sequence of NA12878 genome can be downloaded at <http://sv.gersteinlab.org/NA12878_diploid/NA12878_diploid_2017_jan7/>, and the variants is available at [ftp-trace.ncbi.nlm.nih.gov/giab/ftp/release/NA12878_HG001/](ftp://ftp-trace.ncbi.nlm.nih.gov/giab/ftp/release/NA12878_HG001/).
